# Pesticides as a risk factor for cognitive impairment: Natural substances are expected to become alternative measures to prevent and improve cognitive impairment

**DOI:** 10.3389/fnut.2023.1113099

**Published:** 2023-02-24

**Authors:** Liankui Wen, Xiwen Miao, Jia Ding, Xuewen Tong, Yuzhu Wu, Yang He, Fei Zheng

**Affiliations:** ^1^College of Food Science and Engineering, Jilin Agricultural University, Changchun, China; ^2^National Engineering Research Center for Wheat and Corn Deep Processing, Changchun, China; ^3^Jilin Ginseng Academy, Changchun University of Chinese Medicine, Changchun, China

**Keywords:** cognitive impairment, mechanisms, natural substances, pathways, pesticides

## Abstract

Pesticides are the most effective way to control diseases, insects, weeds, and fungi. The central nervous system (CNS) is damaged by pesticide residues in various ways. By consulting relevant databases, the systemic relationships between the possible mechanisms of pesticides damage to the CNS causing cognitive impairment and related learning and memory pathways networks, as well as the structure–activity relationships between some natural substances (such as polyphenols and vitamins) and the improvement were summarized in this article. The mechanisms of cognitive impairment caused by pesticides are closely related. For example, oxidative stress, mitochondrial dysfunction, and neuroinflammation can constitute three feedback loops that interact and restrict each other. The mechanisms of neurotransmitter abnormalities and intestinal dysfunction also play an important role. The connection between pathways is complex. NMDAR, PI3K/Akt, MAPK, Keap1/Nrf2/ARE, and NF-κB pathways can be connected into a pathway network by targets such as Ras, Akt, and IKK. The reasons for the improvement of natural substances are related to their specific structure, such as polyphenols with different hydroxyl groups. This review’s purpose is to lay a foundation for exploring and developing more natural substances that can effectively improve the cognitive impairment caused by pesticides.

## 1. Introduction

Pesticides are a class of common chemical substances that are widely used in agricultural production to prevent and control diseases, insects, weeds, and fungi. As an indispensable input, pesticides play an important role in agricultural production, so global pesticide production is increasing rapidly. Studies have demonstrated that without the use of pesticide application, fruit yield would be lost by 78%, vegetables by 54%, and cereals by 32% (1). So far, there are many kinds of pesticides, including insecticides, herbicides, and fungicides (2, 3). The active ingredients of most pesticides are divided into two categories: organic (organophosphorus, organic chlorine, organic nitrogen, etc.) and inorganic (copper sulfate, ferrous sulfate, sulfur, etc.) (1). Although pesticides benefit human life to a large extent, they pollute the environment due to their widespread use, and humans are directly or indirectly exposed to pesticides in food, air, soil, water, and plants through the skin, oral cavity, eyes, respiratory tract, and other ways (4, 5), leading to many health problems, such as cancer, respiratory diseases, and neurological disorders. In neurological disorders, damage to learning and memory function is particularly serious.

The brain is the most advanced nerve center that regulates body’s function, and its structural integrity is crucial to cognitive function (6). The realization of spatial learning and memory ability requires the cooperation of different brain regions, in the CNS, the main brain regions closely related to learning and memory are the hippocampus (CA1, CA3, and DG), prefrontal cortex, amygdala, hypothalamus, and striatum (7). Pesticides enter the body through the blood–brain barrier in different ways that cause damage to brain tissue. Moreover, the number and structure of neurons (pyramidal cells, astrocytes, microglia, etc.) are negatively altered, such as incomplete neuron morphology, atrophy, disordered arrangement, unclear cell membrane structure, and death, which have a particularly serious impact on the brain tissue (8). Mechanisms such as oxidative stress, mitochondrial dysfunction, neuroinflammation, neurotransmitter abnormality, and intestinal dysfunction can damage brain tissue by mediating Keap1/Nrf2/ARE, NF-κB, and other signal pathways, resulting in cognitive impairment. However, at present, most of them are the research of a single mechanism and pathway, and the systematic and comprehensive mechanism and pathway network have not been clearly reported. The side effects of preventing and improving cognitive impairment through drugs are relatively large, so natural substances are loved by people. Studies have verified that some natural substances (such as polyphenols and vitamins) can improve the cognitive impairment caused by pesticides; however, there is no final conclusion on how to effectively screen natural substances.

Based on consulting a large number of databases, the mechanisms and cascade pathways of the cognitive impairment caused by pesticides were systematically studied and discussed in this review, and how to screen the natural substances that can effectively improve the cognitive impairment caused by pesticides and the corresponding improvement mechanisms. It is expected to find the connection between them and explore their effects on brain injury and repair caused by pesticides as an organic whole. Those will lay a foundation for finding effective functional factors to improve cognitive impairment caused by pesticide poisoning.

## 2. Mechanisms of cognitive impairment caused by pesticides

### 2.1. Oxidative stress

Because the brain requires high oxygen consumption, low antioxidant activity, and rich polyunsaturated fatty acids, it is very vulnerable to oxidative damage (9). A number of biological studies have shown that oxidative stress is an important risk factor affecting the CNS and causing cognitive impairment (10). Under normal physiological conditions, cellular reactive oxygen species (ROS) levels and the antioxidant system are stable in dynamic equilibrium (11). When the herbicide glyphosate (GLY) and the fungicide Ziram enter the organism and cannot be fully metabolized, they cause lipid peroxidation reactions, leading to an imbalance of oxidants and antioxidants in brain tissue, triggering an excessive accumulation of ROS and further damage to the structure of biological macromolecules such as DNA, lipids, and proteins. It is manifested by an increase in malondialdehyde (MDA) (12) and a decrease in non-enzymatic antioxidants such as the endogenous antioxidant glutathione (GSH) (13). Enzymes associated with oxidative stress, such as superoxide dismutase (SOD) (14), glutathione peroxidase (GSH-Px), catalase (CAT), peroxidase (POD), and heme oxygenase 1 (HO-1), undergo negative changes (15, 16). In addition, changes in GSH-Px expression affect the GSH/oxidized glutathione (GSSG) ratio. ROS can also activate many kinds of immune cells, such as microglia and astrocytes, to release a large number of inflammatory factors, which are associated with neuroinflammatory (17) and induce cholinergic and glutamate toxicity to aggravate oxidative stress (18, 19). These changes cause severe damage to the CNS and are important factors for cognitive impairment.

### 2.2. Mitochondrial dysfunction

The structural integrity of mitochondria is very important for the normal operation of energy metabolism, nerve cell energy synthesis (ATP), and the regulation of calcium and ROS homeostasis, which is the basis of the normal CNS (20). Mitochondria contain the main sites of ROS production, which are also important targets of ROS damage, so oxidative stress damage is a major factor in mitochondrial dysfunction (21). Under normal circumstances, mitochondria also play a role in controlling the dynamic balance of Ca^2+^ concentration. When pesticides enter the organism, ROS accumulates in the brain tissue, which reduces mitochondrial membrane potential, resulting in membrane integrity damage and mitochondrial DNA (mtDNA) release (22). The accumulation of mtDNA mutations also increases the level of ROS, which affects the function of mitochondria. ROS excess also causes mitochondrial Ca^2+^ overload. Once the Ca^2+^ concentration exceeds the physiological threshold, ATP supplied by mitochondria is insufficient, and the mitochondrial function is disordered. This will lead to delayed mitochondrial energy metabolism, as well as potential instability and interruption of neural signal transmission, resulting in severe cell damage. Peroxidation further contributes to the decreased affinity of oxidatively modified cardiolipin for the pro-apoptotic factor Cytochrome C (Cyt-C), prompting the dissociation of Cyt-C from the inner mitochondrial membrane and thus its release into the cytoplasm. For example, the pesticide MCP significantly upregulated ROS, Cyt-C, and lipid peroxide in PC12 cells (23). Cyt-C binds to apoptotic protease activator 1, stimulates the formation of apoptotic complex apoptotic vesicles, and participates in the apoptotic process of activating caspases (24), upregulating the protein and gene expression levels of caspase-3 and caspase-9, and inducing apoptosis and severe neurodegenerative changes. This apoptotic pathway is also regulated by the Bcl family and other related pathways, such as Bcl-2, Bcl-W, Mcl-1, and Bax.

### 2.3. Neuroinflammation

Various pesticides have been shown to elevate inflammatory factors levels, leading to neuroinflammation, neuronal cell death, and cognitive impairment (25). Microglia and astrocytes are the main immune cells in the brain, which are more prone to inflammation (26). Under normal physiological conditions, microglia and astrocytes are in a resting state, maintaining the dynamic balance of the internal environment of the CNS and ensuring the smooth progress of synaptic activities. When the CNS is stimulated by the herbicide paraquat and the pyrethroid bifenthrin (BF) (27, 28), microglia and astrocytes rapidly activate and proliferate, and a tissue repair process is initiated in response to this stimulus (29, 30). Activated microglia, astrocytes, and oxidized mtDNA can all stimulate the NLRP3 inflammasome (31), which is the core of inflammation and an important component of innate immunity. NLRP3 triggers the activation of caspase-1, which can induce apoptosis. Simultaneous activation of NF-κb and other pathways leads to the maturation and secretion of a large number of inflammatory factors such as interleukin-1β (IL-1β), interleukin-6 (IL-6), tumor necrosis factor-α (TNF-α), and cyclooxygenase-2 (COX-2) (32, 33). These inflammatory factors will again lead to the dysfunction of mitochondria, produce ROS, increase the levels of MDA and nitric oxide (NO), and decrease the levels of GSH, CAT, SOD, and GSH-Px (28).

### 2.4. Neurotransmitter abnormalities

Synapses are the key site for information transmission between neurons, and neurotransmitters are crucial for maintaining brain function, as important information transmission substances between nerve cells. Abnormal release and transmission of neurotransmitters can affect the formation of the neurological basis of learning and memory long-term potentiation (LTP) (34) and lead to signal transduction disorders in the brain and ultimately to cognitive impairment.

The neurotransmitters affected by pesticide-induced brain injury are mainly divided into choline, free amino acids, and monoamines. (1) Choline substances, such as acetylcholine (ACh) and acetylcholinesterase (AChE), together constitute the central acetylcholinergic neuron system (35). Normally, in the presynaptic membrane, ACh is synthesized by the reversible transfer of acetyl groups from acetyl-CoA to choline (Ch) catalyzed by the enzyme choline acetyltransferase (ChAT). ACh relies on the acetylcholine transporter (VAChT) for transport to the synaptic vesicle and then released into the synaptic gap. Then binds to the cholinergic receptor of the postsynaptic membrane to form acetylase (HAT) and rapidly sheds acetyl groups automatically to form free AChE, while excess ACh in the synaptic gap is hydrolyzed by AChE to Ch and acetate (36). After monocrotophos (MCP) and other pesticides enter the organism (37), the positively charged part of the pesticides structure binds to the negative moment part of AChE. While the electrophilic phosphoryl group covalently binds to the serine hydroxyl group of the esterolysis site of AChE, inhibiting the activity of AChE. It is difficult to restore to free AChE. As a result of the loss of ability to hydrolyze ACh, ACh released from cholinergic nerve endings accumulated in large quantities. This will adapt changes in the cholinergic receptor, muscarinic receptor (mAChR), and nicotinic receptor (nAChR) (38, 39), resulting in CNS toxicity, disturbances in neural signaling, and cognitive impairment. (2) Free amino acids in the brain also play an important role as neurotransmitters in the cerebral cortex and hippocampus, participate in synaptic excitation transmission, and promote learning and memory formation. Free amino acids can be divided into excitatory amino acids (EAAs) such as glutamic acid (Glu) and N-methyl-D-aspartic acid (NMDA) and inhibitory amino acids (IAAs) such as γ-aminobutyric acid (GABA) and Gly. Under normal circumstances, these neurotransmitters are in a state of dynamic balance to ensure the normal transmission of nerve signals. For example, as an important inhibitory neurotransmitter in the CNS, GABA can antagonize the toxicity of Glu. When the herbicide atrazine and organophosphorus insecticide chlorpyrifos (CPF) enter the organism, the levels of these amino acids fluctuate abnormally, and nerve signal transduction is disordered, affecting the synthesis and regulation of related learning and memory proteins and resulting in brain function impair and cognitive impairment (40, 41). (3) Monoamine neurotransmitters include dopamine (DA), norepinephrine (NE), and 5-hydroxytryptamine (5-HT). They play an important role in the signal transmission of the CNS and have the functions of regulating movement, emotion, sleep, cognition, and memory. Pesticides such as Amitraz, Paclobutrazol, and Profenofos can reduce the expression of monoamine neurotransmitters in the brain tissue (42, 43).

### 2.5. Intestinal dysfunction

It is found that the intestinal microbiota is closely related to the physiological function and disease of the CNS. The intestinal microbiota is linked to the brain through the nervous system, immune system, and endocrine system by neurotransmitters (such as 5-HT, DA, and GABA), immune factors (such as 1L-1β and TNF-α), and endocrine hormones (such as cortisol and corticotropin), thereby directly or indirectly affecting the brain function (44, 45). The accumulation of pesticides can change the composition of intestinal microbiota, promoting the downregulation of beneficial bacteria and overgrowing pathogenic bacteria, which can cause damage to intestinal mucosal and increase intestinal permeability, and allow intestinal bacteria and metabolites to enter the systemic circulation and be recognized by the brain through the nerve, immune, and endocrine pathways, affecting the CNS and inducing nerve injury. Endotoxin lipopolysaccharide (LPS), an important harmful metabolite, is released into the intestinal cavity during proliferation or lysis, activating macrophages and glial cells, increasing NF-κB activity (46), releasing inflammatory factors such as TNF-α and 1L-1β, and inducing inflammation and oxidative stress. However, the levels of beneficial metabolite short-chain fatty acids (SCFAs) decrease (47), affecting brain development and behavior. SCFAs are involved in the regulation of blood–brain barrier permeability and human glial cell homeostasis, and they maintain homeostasis in the CNS (48).

It is found that intestinal microbiota is, indeed, related to brain cognitive function. Aitbali et al. (49) found that the number of the beneficial intestinal microbiota of *Actinobacteria* (*Corynebacterium*), *Firmicutes* (*Lactobacillus*), and *Bacteroidetes* in the gut was decreased after acute (one administration of 0.3 mL), sub-chronic, and chronic exposure (250 or 500 mg/kg/d for 6 and 12 weeks) to GLY-based herbicides. The elevated plus maze and tail suspension test showed that the mouse showed anxiety and depression-like symptoms, indicating that intestinal ecological disorders may lead to neurobehavioral changes. By establishing a mice model of chronic Rotenone-induced Parkinson’s disease (PD), Zhao et al. (50) found that the insecticide rotenone (30 mg/kg/d BW by gavage) could induce intestinal microbiota imbalance and lead to intestinal dysfunction and behavioral disorder. *Akkermansia* and *Desulfovibrio* in feces were increased by 16S rRNA sequencing. Rotenone decreased the expression of tight junction proteins (claudin-1, occludin, and ZO-1), and transmission electron microscopy showed that the intestinal barrier and blood–brain barrier were damaged. The destruction of the intestinal microbiota induced the increase of its metabolite LPS in colon, serum and substantia nigra. LPS promoted the activation of the TLR4/MyD88/NF-κB signaling pathway and enhanced the expression of inflammatory factors (TNF-α, IL-6, IL-1β, iNOS and COX-2), thereby causing damage to neurons. The study showed that rotenone-induced microbiota imbalance may mediate inflammation caused by LPS-TLR4 signaling pathways, thus participating in the occurrence of PD through the microbiota–gut–brain axis. Based on these studies, it can be speculated that intestinal dysfunction may be the key to cognitive impairment caused by pesticides, but there are few related studies at present. Therefore, we think it is necessary to deeply explore the specific mechanism of gut microbiota affecting brain function through information exchange between the gut–brain axis, which may rewrite the definition of a healthy diet and life, and human beings will find new microecological methods to prevent and improve cognitive impairment in pesticides poisoning.

**Figure 1:**
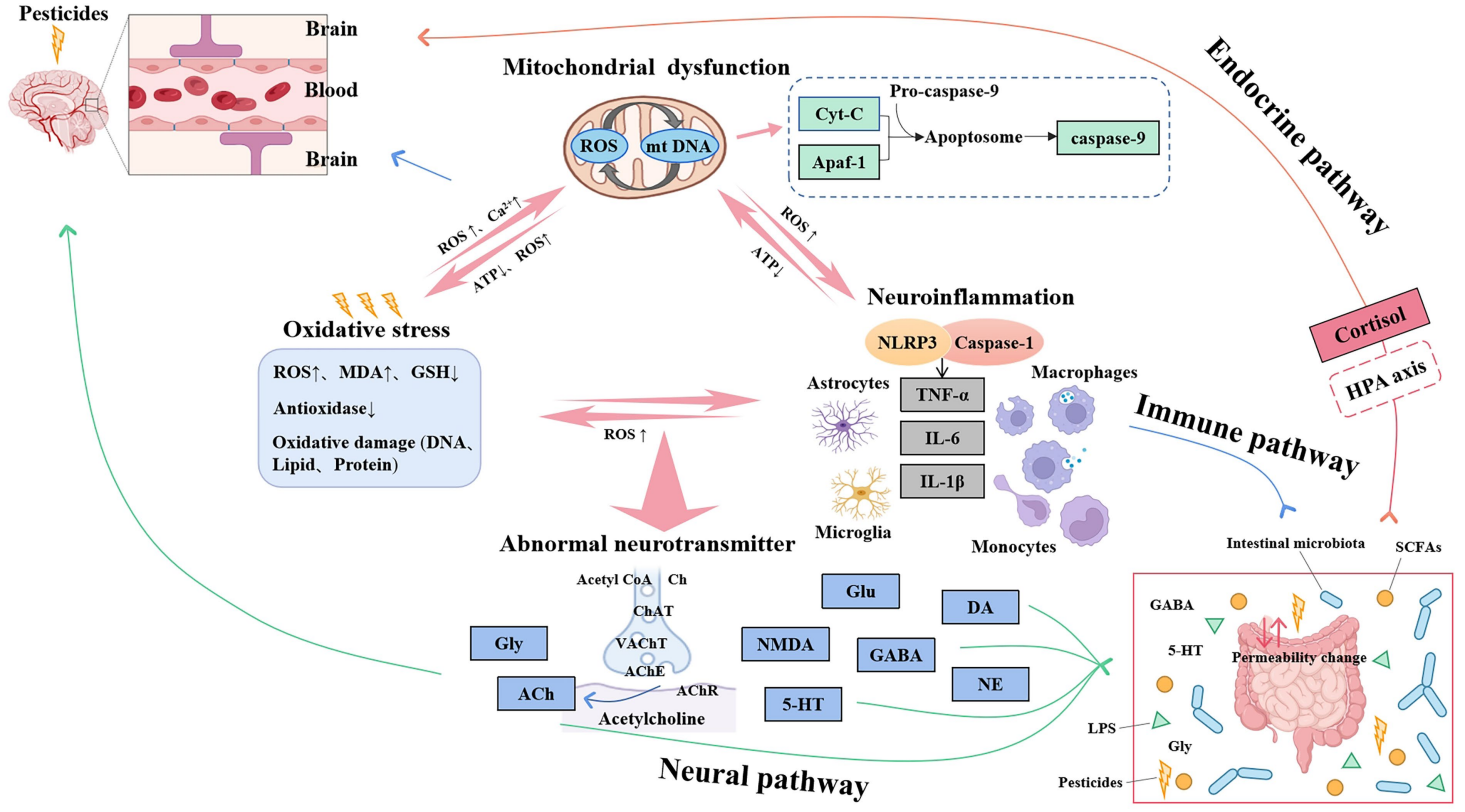
Relationship between mechanisms of pesticides-induced cognitive impairment.

In summary, pesticides do not play a toxic role through a single mechanism, and there are multiple links between the mechanisms (Figure 1). A large amount of ROS is accumulated due to oxidative stress, which leads to the increase of mitochondrial mtDNA and Ca^2+^, and triggers mitochondrial dysfunction. Oxidized mtDNA promotes the activation of NLRP3 inflammasomes, releases a large number of inflammatory factors, and causes neuroinflammatory reactions, forming three feedback loops. Excessive free radicals in oxidative stress can also attack the structure and function of cholinergic neurotransmitter receptors, change cholinesterase activity, and damage the cholinergic system. Oxidative stress affects the expression of Gly; Gly is a constituent amino acid of the endogenous antioxidant reduced Glu in free amino acids. Acetyl-CoA is the raw material for the synthesis of ACh in the cholinergic system, which needs to be synthesized in mitochondria, and Glu is formed by multiple steps in the mitochondria of astrocytes (51). When the supply of ATP from mitochondria is insufficient, it will affect the synthesis of ACh, depolarize the cell membrane, and release a large amount of Ca^2+^-dependent Glu, resulting in cytotoxicity. Immune cells are involved in the expression of Ch acetyltransferase, AChE direct synthetase, and other corresponding cholinergic components. ACh released by immune cells or cholinergic neurons regulates immune function by acting on their receptors in an autocrine/paracrine manner, which can be called the “cholinergic anti-inflammatory pathway” mechanism (52). Therefore, the mechanisms are closely related and inseparable.

However, there are still some shortcomings in the research on the pesticide-induced cognitive impairment mechanism: the mechanism of the toxic effect of different pesticides is not fixed and cannot be fully classified, and it is also unknown whether the structure of pesticides is one of the causes of cognitive impairment. Studies have shown that the action mechanism of organophosphorus pesticides is widely considered to inhibit AChE. The atomic energy of P in organophosphorus pesticides combines with AChE to form phosphoryl cholinesterase, which makes the organism lose the ability to balance ACh, thus causing damage to the cholinergic central nervous system (53). Bipyridine heterocycle herbicide paraquat relies on the transfer of high-energy electrons in its planar bicyclic structure from the mitochondrial electron transfer chain to molecular oxygen to produce toxic ROS, which can induce cognitive impairment through oxidative stress (54). The rest of the pesticides according to their structure also can be divided into organic chlorine, carbamate, and pyrethroid (55, 56). There is no detailed report on whether the mechanism causing cognitive impairment is related to their respective special structures. In addition, a kind of pesticide can damage brain tissue through different mechanisms, but the specific reasons still need a lot of research and verification. At present, many pesticides have been banned in different countries, so it is very important to identify the specific mechanism of cognitive impairment caused by pesticides, and the specific effects of the structural components of pesticides on the CNS and human body. The development of new low-toxicity and high-efficiency pesticides has become an important topic for the global pesticide environment and human safety.

## 3. Related pathways of pesticides affecting learning and memory ability

### 3.1. NMDAR

N-methyl-D-aspartic acid receptor (NMDAR) is a Glu excitatory nerve receptor, which is mainly distributed in the presynaptic and postsynaptic membranes of the cerebral cortex, hippocampus, thalamus, and striatum. It plays an important role in synaptic plasticity and synaptic transmission (57). At resting potential, NMDAR channels are blocked by Mg^2+^, and the NMDAR function is inhibited. Glu and Gly can activate NMDAR to open its channel. At this time, Mg^2+^ flows out of the channel, Ca^2+^ flows in and conducts signal transduction after synapse, which induces the formation of LTP and promotes synaptic plasticity. When pesticides, such as CPF, enter the organism (58), neurons release a large number of Glu, and NMDAR is overactivated, resulting in a Ca^2+^ influx. Ca^2+^-bound calmodulin (CaM) compensation increases, resulting in calmodulin-dependent protein kinase II (CaMKII) expression (59). It also affects the expression of cascade mitogen-activated protein kinase (MAPK), cAMP response element binding protein (CREB), and brain-derived neurotrophic factor (BDNF). NMDAR also affects the normal expression of proteins related to learning and memory such as PSD-95, NR2A, NR2B, and phosphatidylinositol 3 kinase (PI3K) (60). PSD-95 is a key skeleton protein of NMDAR and neuronal nitric oxide synthase (nNOS), which plays an important role in neural development (61). NR2A and NR2B can regulate Ca^2+^ permeability and Mg^2+^ sensitivity, and induce and maintain LTP.

### 3.2. PI3K/Akt

A number of studies suggest that PI3K/Akt is an important oxidative signaling pathway and is closely related to learning and memory ability (62). PI3K/Akt signaling pathway is associated with multiple cascade pathways to maintain cellular homeostasis through antioxidant, anti-inflammation, and other mechanisms. When cells are damaged by oxidative stress caused by pesticides, excessive production of ROS over-regulates the level of PI3K (63). PI3K promotes the production of phosphatidylinositol triphosphate (PIP3), which then activates the high expression of Akt activated by PIP3 (64), affecting the normal regulation of downstream signaling molecules such as glycogen synthase kinase-3β (GSK3β), Nrf2, NF-κB, and mammalian rapamycin target protein (mTOR). For example, rotenone mediates the PI3K/Akt/mTOR pathway leading to brain injury and motor dysfunction in mice, and multiple impairments including the degeneration of dopaminergic neurons, decreased DA and GSH, and elevated MDA are also present (65). Mohammadzadeh et al. (66) found that the organophosphorus pesticide malathion induced spatial learning and memory impairment, activated GSK-3β, and inhibited protein phosphatase 2A (PP2A) in rats, while also increasing the levels of TNF-α and IL-6. GSK3β of an appropriate amount widely exists in neurons and glial cells of the brain and plays an important role in the regulation of cognitive function (67). Instead, excess GSK3β inhibits the activity of the antioxidant transcription factor Nrf2, preventing Nrf2 from playing a normal role in regulating cognitive function.

### 3.3. MAPK

Mitogen-activated protein kinase is a group of serine–threonine protein kinases that can be activated by cytokines, neurotransmitters, hormones, and cell stress, which is an important information transmission chain (68). The main subclasses of MAPKs are extracellular regulated protein kinases (ERK1/2), c-jun terminal kinase (JNK), and p38 kinase (p38/MAPK) (69). After MAPKs activation, ERK1/2 is preferentially activated by growth factors, while p38/MAPK and JNK are particularly sensitive to environmental stress and cytokines (such as TNF-α) (70). They participate in the regulation of several inflammatory transcription factors (such as NF-κB) through phosphorylation. ERK1/2 is mainly responsible for cell growth and differentiation, and its upstream signal is the famous Ras/Raf protein (71). Stimulated by pesticides such as CPF (72), Ras is activated by binding to guanosine triphosphate, thus phosphorylating and activating Raf. Raf reactivates MEK1/2, and phosphorylated MEK1/2 activates ERK1/2. Activated ERK1/2 phosphorylates ribosomal S6 protein kinase (RSK)-like protein kinase substrates and co-enters the nucleus to promote the phosphorylation of important transcription factors such as CREB and regulate the transcriptional expression of Bcl-2, c-fos, c-myc, c-jun, Egrl, and other genes (73). p38/MAPK may be involved in the post-transcriptional regulation of COX-2, iNOS, and TNF-α (74). JNK is similar to p38 in function. The damage to brain tissue by pesticides may be related to three important branch pathways of MAPK, ERK1/2, p38/MAPK, and JNK.

### 3.4. Keap1/Nrf2/ARE

Keap1/Nrf2/ARE signaling pathway plays an important role in cellular resistance to endogenous or exogenous oxidative stress and inflammation (75). Normal cells resist the interference of foreign substances through the antioxidant and anti-inflammation effects produced by the Keap1/Nrf2/ARE signaling pathway and its downstream gene activation. Increased intracellular ROS levels after pesticide intervention can promote the dissociation of the important transcription factor Nrf2 and the specific receptor Keap1 that regulates the anti-oxidative stress response in the body (76). Nrf2 enters the nucleus and forms a heterodimer with the small Maf protein, activating the antioxidant response element (ARE) to regulate the expression of phase II detoxification enzyme genes (NQO1 and GSTs) and antioxidant enzyme genes (SOD, HO-1, and CAT) (77, 78). For example, cypermethrin could inhibit the Nrf2/ARE pathway and decrease the expression of HO-1 and NQO1 (79). Nrf2 also regulates the GSH redox system, mainly regulating the expression of γ-GCS, and γ-GCS is the rate-limiting enzyme of GSH biosynthesis (80). Nrf2 also regulates the regeneration of GSH by controlling GSH-Px expression, GSH can be oxidized to GSSG by GSH-Px, and GR regenerates GSSG generated by GSH-Px to GSH (81). At the same time, this pathway increases the ability of cells to react to ROS and resistance to oxidative stress markers such as lipid peroxidation products, thus having a protective effect on multiple organs.

### 3.5. NF-κB

NF-κB is a multiple nuclear transcription-inducing factor that exists in almost all animal cells. It participates in the response of cells to external stimuli. It plays a key role in cellular immune response and inflammatory response and is closely related to learning and memory (82). NF-κB protein activity is activated by the phosphorylation of IκB kinase (IKK). In the resting state, NF-κB binds to IκB in the cytoplasm to form an inert complex that stays in the cytoplasm. When cells are stimulated by pesticide-induced inflammation signals, IKK is first activated (83). Then IKK phosphorylates the intracellular IκB protein. The phosphorylated IκB protein is, in turn, degraded by proteases, thus releasing NF-κB (84). The activated NF-κB enters the nucleus, binds to genes with NF-κB binding sites, and thus initiates the transcription process, inducing an increase in the levels of inflammatory factors such as TNF-α, IL-1β, and apoptotic factors such as Bcl-2 to affect learning and memory (85). Michel et al. (86) found that rotenone-induced NF-κB transcription increased the expression of iNOS and COX-2 in the rat’s midbrain and striatum. Rotenone also increased caspase-3 and α-synuclein activity, as well as the Bax/Bcl-2 ratio in the striatum to induce apoptosis.

**Figure 2:**
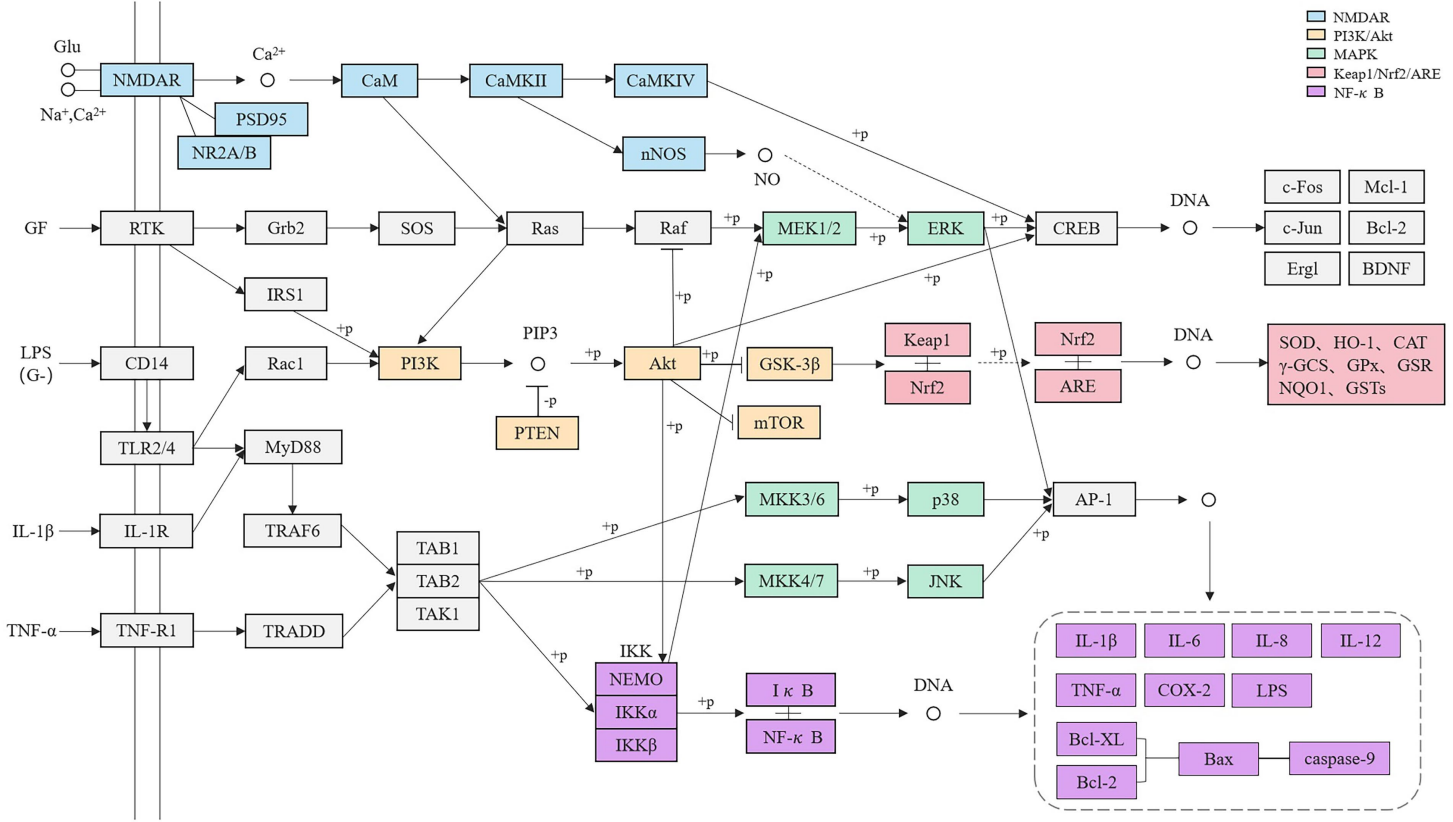
Related pathways network of pesticides poisoning affecting learning and memory ability.

After sorting out, it is found that each pathway is not independent but has interconnected targets, which establishes a huge pathways system (Figure 2) and together play a regulatory role in the process of pesticide-induced brain tissue injury. It is assumed that under the stimulation of pesticides, a large amount of Glu accumulates, activating that NMDAR pathway, resulting in Ca^2+^ influx and activation of downstream PI3K/Akt and MAPK signaling pathways through CaM and Ras. The γ subtype of PI3K plays a key role in NMDAR receptor-mediated synaptic plasticity and hippocampal-dependent learning and memory ability. PI3K can activate CREB related to the ERK pathway, and its over-activation can reduce the probability of ERK entering the nucleus. As an important injury junction, Akt can attenuate the expression of the Nrf2 nuclear transcription factor in the Keap1/Nrf2/ARE pathway, and reduce the activities of antioxidant enzymes such as SOD. It can also promote the initiation of the NF-κB pathway and release a large number of inflammatory factors such as TNF-α and IL-1β. NF-κB can also initiate the Bcl apoptosis pathway, resulting in cell damage and death. All three branches of MAPK will promote the production of inflammatory factors finally, and it is believed that MAPK and NF-κB pathways are inextricably linked. By constructing the pathway network, it is not difficult to guess that the related signaling pathways in pesticide-induced cognitive impairment are an organic whole and closely related to the action mechanism. These pathways together lead to cognitive impairment caused by pesticide poisoning.

## 4. Natural substances for improving cognitive impairment induced by pesticides

### 4.1. Polyphenols

#### 4.1.1. Kaempferol

Kaempferol has a neuroprotective effect on brain damage caused by neurodegenerative diseases (87, 88). Hussein et al. (89) used kaempferol (21 mg/kg/d BW by injection) to interfere with the rat model of neurodegenerative disease induced by CPF (18 mg/kg/d BW by injection) for 14 days. Through behavioral experiments, it was found that kaempferol could reverse CPF exposure and improve the spatial and non-spatial memory ability of rats. Kaempferol may be able to prevent neuronal degeneration, atrophy, apoptosis, and edema in the CA1, CA3, and DG regions of the hippocampus. Kaempferol could downregulate the contents of ROS and MDA and enhance the activities of SOD, GSH-Px, and CAT. It also could regulate and control the GSK3β-Nrf2 pathway (by inhibiting the activation of GSK-3β and decreasing nuclear Nrf2), alleviating and inhibiting the oxidative stress induced by CPF. At the same time, the activity of AChE was also inhibited.

Kaempferol is a flavonol compound with a molecular weight (MW) of 286.236. Its structure is C ring (one hydroxyl, one double bond, and one ketone group), aromatic A ring (two hydroxyl groups), and aromatic B ring (one hydroxyl group). One study compared the improvement of cognitive impairment between kaempferol and two other flavonol substances with similar structures (A ring and C ring are the same), namely galangin (there is no hydroxyl group on the B ring) and myricetin (there are three hydroxyl groups on the B ring). The results showed that the increase of hydroxyl number on the B ring significantly upregulated the expression of the ERK1/2-CREB pathway in brain tissues of cognitively impaired mice, thus exerting neuroprotective effects. At the same time, kaempferol and myricetin can significantly improve the spatial learning and memory ability of mice, while galangin has no significant improvement effect. However, all three inhibited oxidative stress, and there was no significant difference (90). This suggests that antioxidants may not be the only mechanism by which natural substances exert neuroprotective effects, and the specific reasons need to be further confirmed. In addition, some studies have shown that the antioxidant activity of myricetin depends not only on the hydroxyl group on the B ring, but also on the hydroxyl group and double bond on the C ring (91), so it can be speculated that the antioxidant effect of galangin may also depend on the hydroxyl group and double bond on the C ring.

#### 4.1.2. Quercetin

Quercetin has multiple biological effects and a good puzzle effect (92, 93). Beghoul et al. (94) intervened rats with quercetin (5 mg/kg/d BW by gavage) and synthetic insecticide pyrethroid (3.2 mg/kg/d BW Bifenthrin; 2.6 mg/kg/d BW deltamethrin by gavage) and found that quercetin could prevent pyrethroid-induced mitochondrial swelling in the hippocampus, striatum, cortex, and cerebellum. It could also play an antioxidant role by increasing the level of GSH and the activities of CAT and GST, reducing the level of MDA. Fereidouni et al. (95) established a neurodegenerative mice model with CPF (13.5 mg/kg/d BW) and intervened with quercetin (50 mg/kg/d BW). It was found that quercetin could reduce cell death by regulating the expression of Bax, Bcl-2, Cyt-C, caspase-8, and caspase-9 in the brain tissue, thus playing a neuroprotective effect. Josiah et al. (96) intervened PD rats induced by rotenone (1.5 mg/kg/d BW by injection) with quercetin (20 mg/kg/d BW significantly). The results showed that quercetin could improve the neuronal density depletion caused by rotenone toxicity, optimize the disordered DA metabolism, and inhibit the expression of NF-κB, IL-1β, TNF-α, IκB, and pro-apoptotic gene p53 in the striatum.

Quercetin (MW: 302.236) is a flavonol compound. Its C ring and aromatic A ring are the same as kaempferol and myricetin; the difference is that there are two adjacent hydroxyl groups on the aromatic B ring (91). A simple hypothesis is that the ability of quercetin to improve cognitive impairment is intermediate between kaempferol and myricetin, but it cannot be ruled out that the arrangement of hydroxyl groups will affect its improvement effect. At the same time, it is unknown whether their improvement ability is limited to specific mechanisms and pathways, which needs a lot of research to verify.

#### 4.1.3. Myricetin

It has protective and therapeutic effects on cancers, diabetes, PD, and AD (97, 98). Dhanraj et al. (99) used myricetin (314 mM) to interfere the PD model of Drosophila induced by the rotenone (250, 500, 750, and 1,000 μm). Through a variety of behavioral experiments, it was found that myricetin could improve neuromuscular incoordination and memory ability induced by rotenone. The neuron clusters in Drosophila brain tissue were repaired. The levels of DA, GSH, SOD, CAT, GSH-Px, and Bcl-2 increased, and the levels of Bax, caspase-3, caspase-9, Cyt-C, and lipid peroxidation decreased. The joint evaluation of these phenomena showed that myricetin had a good neuroprotective effect and could alleviate the symptoms of cognitive impairment in PD.

Myricetin (MW: 318.235) is a flavonol compound. The study found that the hydroxyl group of myricetin B ring and C ring can regulate the expression of PI3K, and the ketone group and double bond on the C ring can inhibit the anti-inflammatory activity of COX-2 expression (91), which may also be an important reason why myricetin can improve cognitive impairment.

#### 4.1.4. Baicalein

Baicalein has been used for the treatment of hypertension, cardiovascular disease, cancer, and cognitive impairment. Li et al. (100) found that baicalein could inhibit Rotenone-induced (2 μm) apoptosis by exploring the molecular mechanism of the effect of baicalein (40 μm) on PC12 cells and rats’ brain mitochondria *in vitro*. It inhibited the accumulation of ROS, ATP deficiency, caspase-3 activation, mitochondrial membrane potential dissipation, and mitochondrial swelling, which suggested that baicalein may be a mitochondrial-targeted antioxidant and has a neuroprotective effect on rotenone-induced neurotoxicity.

Baicalein (MW: 270.237) is a flavonoid. Its C ring contains a double bond and a ketone group. The aromatic A ring has three hydroxyl groups and the B ring does not contain hydroxyl groups (101). It can speculate that baicalein has antioxidant and neuroprotective effects because of the double bond and ketone groups on its C ring and extra one hydroxyl group on the A ring, or perhaps in fact the hydroxyl group on the A ring is the most important factor to exert activity, but this still needs some comparative tests to verify.

#### 4.1.5. Luteolin

Luteolin, with a variety of biological activities (102), has been shown to well improve pesticide-induced cognitive impairment. Seydi et al. (103) found that luteolin could protect the brain from oxidative stress and mitochondrial toxicity induced by fipronil (FPN) (6, 12, 24 μm) in rats. Luteolin (25 μm) could reduce brain mitochondrial swelling by reducing the levels of ROS and MDA and increasing the levels of GSH and ATP. The results showed that luteolin had a significant protective effect on FPN-induced brain mitochondrial neurotoxicity by improving oxidative stress and mitochondrial dysfunction.

The flavonoid compound luteolin (MW: 286.236) is similar to baicalein, with the C ring (one double bond and one ketone group) and A ring (three hydroxyl groups), but its aromatic B ring has two ortho hydroxyl groups more than baicalein. These functional groups may be responsible for conferring its neuroprotective effects (104, 105). It can be speculated that luteolin is more active than baicalein in certain mechanisms and pathways. There are no hydroxyl groups on the C ring of luteolin and baicalein, indicating that the hydroxyl groups on the C ring are not the decisive factor for antioxidant and neuroprotective effects.

#### 4.1.6. Curcumin

Curcumin has various biological activities such as anti-inflammation and anti-oxidation (106, 107). Hasan et al. (108) found that curcumin (30 mg/kg/d BW by gavage) had a protective effect on rotenone-induced (3 mg/kg/d BW by gavage) cerebellar toxicity in mice for 60 days. Curcumin could improve the symptoms of walking difficulty caused by rotenone, such as shortening of front and rear limb stride and widening of the base. It also reduced the production of NO, enhanced the activities of GSH, SOD, and CAT, and significantly increased the activity of AChE. Curcumin could protect mice from irregular and damaged Purkinje cells and peripheral vacuolization caused by rotenone exposure.

Small molecule (MW: 368.39) curcumin is a phenolic compound. It contains many phenolic hydroxyl groups, ketone groups, and double bonds; these make it have an antioxidant effect. It has also been found that the olefin saturation of the linkage bond at the seventh C atom of curcumin and the 4-hydroxyphenyl in its structure affect the anti-inflammatory effect of curcumin (109). These structural features may affect the ameliorative effect of curcumin on cognitive impairment.

#### 4.1.7. Anthocyanins

Anthocyanins are flavonoid compounds, and their R_1_ and R_2_ of the B ring can be replaced by different substituents (−OH, −OCH3), resulting in six common categories. The MW of anthocyanins is 287.240~—. Large MW anthocyanins have to be metabolized into small molecules in order to be absorbed and improved. Anthocyanins are powerful antioxidants that protect the eyes and liver and prevent and treat diabetes, cancer, and CNS diseases (110, 111). In recent years, much attention has been paid to the effect of anthocyanins on improving CNS diseases and many studies have been carried out.

Although there are no detailed reports on anthocyanins improving cognitive impairment in pesticides models, the results of some other models have shown that anthocyanins are a safe therapeutic agent that can alleviate brain tissue damage and improve cognitive impairment caused by oxidative stress, neuroinflammation, etc. (112), suggesting that anthocyanins have the potential to improve the cognitive impairment caused by pesticides.

### 4.2. Terpenoid

Carnosic acid (CA) has many biological activities, such as inhibiting oxidative stress and inflammation (113, 114), and it has a good protective effect in the experimental model of neurodegenerative diseases. De Oliveira et al. (115) used CA (0.1–2 μm) to intervene with SH-SY5Y cells in the PD model induced by Paraquat (100 μm) and found that CA could be used as a neuroprotective agent to prevent the effects of Paraquat on cell viability and redox parameters, increase the contents of total GSH and mitochondrial GSH in SH-SY5Y cells, reduce the production of ROS, RNS, and the toxicity to mitochondrial function. At the same time, inhibition of the PI3K/Akt pathway or silencing of Nrf2 expression by LY294002 partially blocked the improvement of CA. The results showed that CA could activate Nrf2 by regulating PI3K/Akt pathway, resulting in an increase in the level of antioxidant enzymes and a neuroprotective effect. AlKahtane et al. (116) studied the improving effect of CA (60 mg/kg/d BW by gavage) on mice brain and eye cytotoxicity induced by CPF (12 mg/kg/d BW by gavage). It was found that CA could reduce the contents of MDA and NO in the brain and eye tissue, decrease the levels of IL-1β, IL-6, and TNF-α in serum, and increase the level of AChE in serum and the levels of GSH, GSH-Px, SOD, and CAT in the brain.

Carnosic acid is a natural phenolic diterpene with an MW of 332.434. It is speculated that the two hydroxyl groups on the benzene ring in its molecule are one of the key reasons for its antioxidant activity. However, whether other characteristics of CA structure are related to the improvement of cognitive impairment needs to be verified.

### 4.3. Biological hormones

#### 4.3.1. Melatonin

Melatonin (MEL) is an important nervous system regulator and antioxidant (117, 118). Sharma et al. (119) studied the effect of MEL (5 mg/kg/d BW by injection) on the regulation and improvement of cognitive impairment induced by phosphoramide (PHOS) (1.74 mg/kg/d BW by gavage) in rats. The results showed that intervention with MEL could antagonize the behavioral effect. MEL could significantly reduce the level of MDA in brain tissue and alleviate the effect of oxidative stress on the brain. Mehta et al. (120) studied the regulatory effect of MEL on the cognitive impairment induced by propoxur (10 mg/kg/d BW by gavage) and found that MEL (50 mg/kg/d BW by injection) could inhibit the increase of MDA and the decline of brain GSH and CAT activities in the brain induced by propoxur. The results showed that MEL had the potential to alleviate the cognitive impairment and oxidative stress caused by propoxur and other poisons in the brain.

Melatonin (MW: 232.278), also known as N-acetyl-5-methoxytryptamine, is the synthetic material for the neurotransmitter 5-HT. (121). It can be well speculated that MEL will be metabolized to 5-HT again in human body, thus improving cognitive impairment. MEL also contains acetyl (CH3–CO−) and methoxy (CH3O−) in its structure, which may also be related to the improvement effect, but the specific mechanism still needs to be verified.

#### 4.3.2. Progesterone

Progesterone (PROG) is an important female hormone, which can protect the fetus, promote pregnancy, and regulate the body temperature center, and is also an important regulator of the nervous system function (122, 123). Sharma et al. (124) found that PROG (15 mg/kg/d BW by injection) could improve the behavioral cognitive impairment induced by acute or chronic PHOS (1.74 mg/kg/d BW by gavage) exposure in rats. PROG could decrease thiobarbituric acid reactive substance (TBARS) and increase the non-protein sulfhydryl group (NP-SH) in the brain induced by PHOS. Therefore, the results showed that PROG could alleviate the cognitive impairment and oxidative stress induced by PHOS in the brain.

Progesterone (MW: 314.462) is a neurosteroid hormone secreted by the ovary. Its structure includes a ketone group and a double bond. This may be the reason why it is easier to combine with the oxygen-free radicals caused by pesticides to avoid oxidative stress in the human body, thus producing neuroprotective effects (125).

### 4.4. Vitamins

#### 4.4.1. Vitamin B6

Vitamin B6 (VB6) can be used to prevent and treat lip splitting, seborrheic dermatitis, and diabetic vascular complications. In recent years, it has been found that VB6 can be used to prevent and treat CNS diseases (126). Li et al. (127) used VB6 (50 mg/kg/d BW by gavage) to interfere with the vascular dementia model rats induced by isocarbophos (0.5 mg/kg/d BW by gavage) and found that VB6 could improve the behavior retardation and memory impairment. VB6 could improve the disappearance, atrophy, disorder, and incomplete and unclear structure of neurons in DG, CA1, and CA3 regions of the hippocampus, inhibit the proliferation of inflammatory cells and astrocytes, and restore reduced cerebral arterial blood flow. VB6 also increased the levels of NR2B, PSD-95, and CaMKII in the rats’ hippocampus. It was concluded that VB6 could activate the NMDAR signal, and its beneficial effect on vascular dementia can be attributed to the regulation of NR2B, PSD95, and CaMKII proteins in the pathway.

VB6 (MW: 231.144) is a water-soluble vitamin; the three hydroxyl groups in the structure endow it with antioxidants and other activities. VB6 mainly reacts with nearly 100 enzymes in the human body in the form of pyridoxal phosphate (PLP) and exerts biological effects. For example, it can participate in the synthesis of neurotransmitters (5-HT, DA, NE, and GABA) (128). These may be the reason why VB6 can effectively improve cognitive impairment, but it still needs to be verified through research.

#### 4.4.2. Vitamin C

Vitamin C (VC) is a natural strong antioxidant and cofactor of various enzymes, thus improving various diseases. Ambali et al. (129) intervened in CPF-induced (10.6 mg/kg/d BW by gavage) cognitive impairment rats with VC and found that VC (100 mg/kg/d BW by gavage) could improve neuromuscular disharmony and neurobehavioral defects. VC could regulate AChE activity and significantly reduce MDA content to improve cognitive impairment. Suke et al. (130) found VC (100 mg/kg/d BW by gavage) had a significant protective effect on behavioral memory by PHOS (1.74 mg/kg/d BW by gavage) induced cognitive impairment, and it could regulate the oxidative stress indexes (MDA, PC, and GSH).

Vitamin C (MW: 176.124), also known as ascorbic acid, is a water-soluble vitamin containing hydroxyl and carbonyl groups (C=O−). Hydroxyl and o-carbonyl groups can form hydrogen bonds to participate in the hydroxylation reaction, thereby promoting the synthesis of neurotransmitters (5-HT, NE). The structural characteristics of VC make it have strong antioxidant and neuroprotective effects (131).

#### 4.4.3. Vitamin E

Vitamin E (VE) can promote the secretion of sex hormones, improve fertility and blood circulation, effectively combat free radicals, and inhibit the production of lipid peroxidation. In recent years, more and more attention has been paid to its improvement in CNS diseases (132). Kosta et al. (133) used VE (125 mg/kg/d BW by gavage) in rats with cognitive impairment induced by PHOS (1.74 mg/kg/d BW by gavage). Through behavioral experiments, it was found that VE could improve memory ability, significantly regulate the levels of MDA and NP-SH, and inhibit oxidative stress.

Vitamin E is a fat-soluble vitamin antioxidant with MW is 430.706. Its phenolic hydroxyl group is the active group. The hydroxyl group may provide a hydrogen atom to reduce ROS levels and exert antioxidant activity, thereby participating in the improvement of cognitive impairment. It was speculated that the number and position of methyl groups on the benzene ring, the side chain, and the stereo structure in the molecule also affected the activity.

Based on the analysis of natural substances that can improve the cognitive impairment caused by pesticides (Table 1), it was found that first, the molecular weight of these natural substances is very small (MW: 150~450), which may be one of the reasons why they are easily able to cross the blood–brain barrier to play an improving role. Second, it is found that the improvement effect of these natural substances is affected by the structure. Different substituents (OH−, CH3–CO−, CH3O−, C=O−, and C=C−) in the molecular structure may all be the reasons for the improvement of natural substances, but which structure improvement effect is better still needs to be verified. In addition, some studies have shown that the increase in the number of hydroxyl groups on the B ring of flavonol is beneficial to neuroprotection, but only limited to the study of specific pathways, further research on the other mechanisms is still necessary. More importantly, flavonoids without hydroxyl groups on the B and C rings also have improvement effects. It is speculated that their active sites may be double bonds and ketones on the C ring, as well as hydroxyl groups on the A ring. Therefore, there are specific effects of different types, positions, and amounts of substituents on the A, B, and C rings of flavonoid compounds on the improvement effect are needed to be verified by a large number of studies, which will facilitate a more accurate screening of effective natural substances. Multivitamins can improve cognitive impairment (134, 135). It is speculated that their common hydroxyl structure guarantees their antioxidant activity. Another interesting discovery is that water solubility or fat solubility have no effect on their functions; they are all well absorbed by the human body. The improvement effect of natural substances on pesticide cognitive impairment is also affected by the classification of natural substances, pesticide types and exposure doses, experimental models (animals, cells, and tissues), and intervention methods (gavage or injection; Table 1). However, the intake of natural substances such as polyphenols and vitamins in the human daily diet is affected by such factors as dietary habits, geographical region, and age (136). Therefore, it is necessary to assess whether daily dietary intake can improve the contact injury caused by pesticides, and reasonably guide the intake of natural substances, so as to effectively improve the corresponding injury in the future.

**Table 1:** Effects and characteristics of natural substances on pesticide-induced cognitive impairment.

Classify	Natural substances	Molecular weights	Constitutional formulas	Models (methods)	Predict pathways	Related regulatory molecules	Mechanisms	References
Polyphenols	Kaempferol	286.236	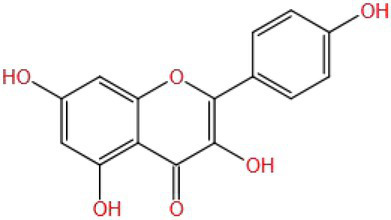	Rat (CPF:18 mg/kg/d; Kaempferol: 21 mg/kg/d)	PI3K/Akt, Keap1/Nrf2/ARE	ROS, MDA, SOD, GSH-Px, CAT, GSK-3β, Nrf2, AChE	Inhibit oxidative stress and regulate neurotransmitter abnormality	(89)
	Quercetin	302.236	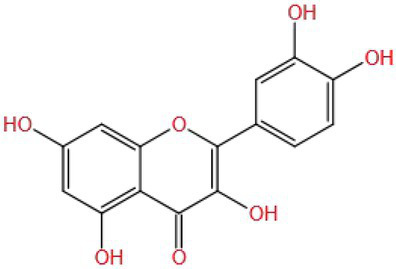	Rat (Bifenthrin: 3.2 mg/kg/d + Deltamethrin 2.6 mg/kg/d; Quercetin: 5 mg/kg/d); Mice (CPF: 13.5 mg/kg/d; Quercetin: 50 mg/kg/d); Rat (Rotenone: 1.5 mg/kg/d; Quercetin: 20 mg/kg/d)	Keap1/Nrf2/ARE, NF-κB	CAT, GSH, MDA, GST, Bax, Bcl-2, Cyt-C, caspase-8, caspase-9, NF-κB, IκB, IL-1β, TNF-α, p53	Inhibit oxidative stress, inhibiting inflammation and improve mitochondrial dysfunction	(94–96)
	Myricetin	318.235	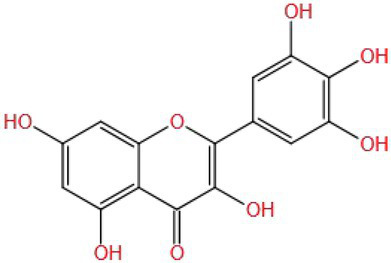	Drosophila (Rotenone: 314 mM; Myricetin: 500 μm)	Keap1/Nrf2/ARE	DA, GSH, SOD, GSH-Px, CAT, Bcl-2, Bax	Inhibit oxidative stress, inhibiting inflammation and improve mitochondrial dysfunction	(99)
	Baicalein	270.237	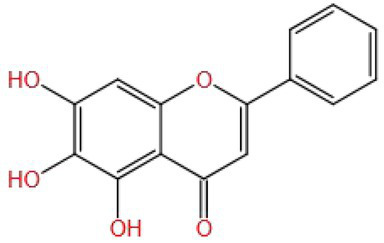	PC12 cell/rat brain mitochondria (Rotenone: 2 μm; Baicalein: 40 μm)	Keap1/Nrf2/ARE	ROS, caspase-3, ATP	Inhibit oxidative stress and improve mitochondrial dysfunction	(100)
	Luteolin	286.236	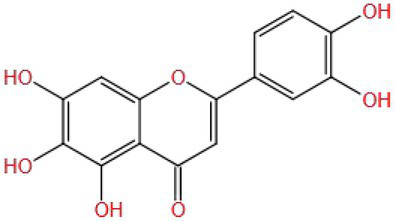	Rat (FPN: 24 μm; Luteolin: 25 μm)	Keap1/Nrf2/ARE	ROS, MDA, GSH, ATP	Inhibit oxidative stress	(103)
	Curcumin	368.39	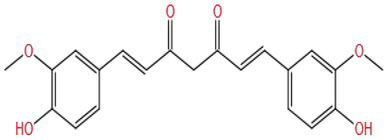	Mice (Rotenone: 3 mg/kg/d; Curcumin: 30 mg/kg/d)	Keap1/Nrf2/ARE	NO, GSH, SOD, CAT, AChE	Inhibit oxidative stress and regulate neurotransmitter abnormality	(108)
Terpenoid	Carnosic acid	332.434	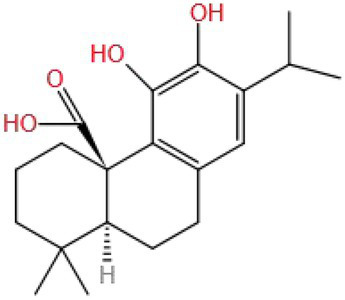	SH-SY5Y cell (Paraquat: 100 μm; CA: 2 μm); Mice (CPF: 12 mg/kg/d; CA: 60 mg/kg/d)	PI3K/Akt,Keap1/Nrf2/ARE, NF-κB	PI3K, Akt, Nrf2, MDA, NO, IL-1β, IL-6, TNF-α, AChE, GSH, GSH-Px, CAT	Inhibit oxidative stress, inhibit inflammation and regulate neurotransmitter abnormality	(115, 116)
Biological hormones	Melatonin	232.278	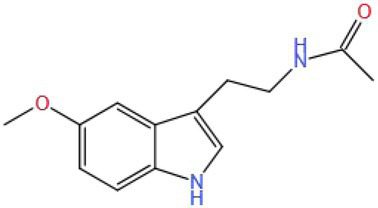	Rat (PHOS: 1.74 mg/kg/d; MEL: 5 mg/kg/d); (Propoxur: 10 mg/kg/d; MEL: 50 mg/kg/d)	Keap1/Nrf2/ARE	MDA, NP-SH, GSH, CAT	Inhibit oxidative stress	(119, 120)
	Progesterone	314.462	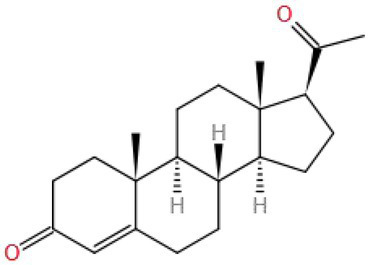	Rat (PHOS: 1.74 mg/kg/d; PROG: 15 mg/kg/d)		TBARS, NP-SH	Inhibit oxidative stress	(124)
Vitamins	Vitamin B6	231.144	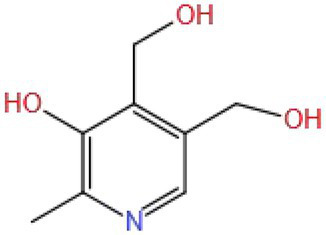	Rat (Isocarbophos: 0.5 mg/kg/d; VB6: 50 mg/kg/d)	NMDAR	NR2B, PSD-95, CaMKII	Inhibit inflammation, regulate neurotransmitter abnormality	(127)
	Vitamin C	176.124	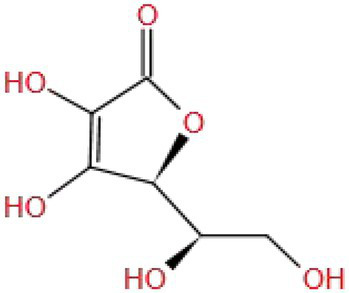	Rat (CPF: 10.6 mg/kg/d; VC: 100 mg/kg/d); (PHOS: 1.74 mg/kg/d; VC: 100 mg/kg/d)	Keap1/Nrf2/ARE	MDA, PC, GSH, AChE	Inhibit oxidative stress and regulate neurotransmitter abnormality	(129, 130)
	Vitamin E	430.706	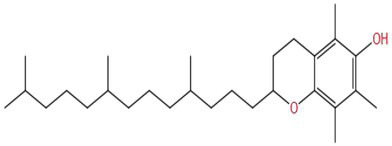	Rat (PHOS: 1.74 mg/kg/d; VE: 125 mg/kg/d)		MDA, NP-SH	Inhibit oxidative stress	(133)

## 5. Conclusion

Pesticide poisoning has seriously increased the risk of CNS diseases and become a serious potential safety hazard in people’s daily life. It was summarized that pesticides could damage brain tissue through action mechanisms such as oxidative stress, mitochondrial dysfunction, neuroinflammation, neurotransmitter abnormalities, and intestinal dysfunction, and these mechanisms were interrelated and had feedback effects. For example, the excessive accumulation of ROS in oxidative stress could lead to the increase of mitochondrial mtDNA and Ca^2+^, leading to mitochondrial dysfunction. The mutation accumulation of mitochondrial mtDNA and the decrease of ATP would also increase ROS level and aggravate oxidative stress. Pesticide-induced cognitive impairment is also accompanied by the regulation of NMDAR, PI3K/Akt, MAPK, Keap1/Nrf2/ARE, NF-κB, and other multiple learning and memory pathways, which can be formed into an organic whole through the crosslinking targets such as RAS, Akt, and IKK. In this article, the characteristics of some natural substances that improve the cognitive impairment of different pesticides were also summarized. It is found that polyphenols, biological hormones, and vitamins are functional factors that can play a good role in improvement. Most of the molecular weights of these natural substances are about 150~450 Da, which may be one reason why they are very suitable for biological activity across the blood–brain barrier. Second, the location and number of structural functional groups (OH−, CH3−CO−, CH3O−, C=O−, and C=C−) in natural substances may affect their efficacy in improving cognitive impairment. Our speculations may be used to screen natural substances with good neuroprotective effects. For example, the increase of the number of hydroxyl groups in the structure of natural substances is beneficial to the enhancement of the antioxidant effect, which makes it play a better role in improving cognitive impairment. Finally, the mechanisms and related pathways of pesticide-induced cognitive impairment are not limited to those presented in this article. The research on the improvement mechanisms of natural substances is not deep enough, and our speculation needs to be verified and supplemented by a large number of studies in the future. More progress will be beneficial to the research and development of healthy food for the prevention and alleviation of cognitive impairment diseases, thus promoting the healthy development of mankind.

## Author contributions

LW and XM: investigation and formal analysis, writing – original draft, review and editing. JD: formal analysis and editing. XT: investigation and editing. YH: conceptualization, project administration, and writing – review and editing. YW and FZ: conceptualization, supervision, and formal analysis. All authors contributed to the article and approved the submitted version.

## Funding

This research was funded by the Jilin Provincial Science and Technology Department, grant number: 20210202105NC and 20210101043JC.

## Conflict of interest

The authors declare that the research was conducted in the absence of any commercial or financial relationships that could be construed as a potential conflict of interest.

## Publisher’s note

All claims expressed in this article are solely those of the authors and do not necessarily represent those of their affiliated organizations, or those of the publisher, the editors and the reviewers. Any product that may be evaluated in this article, or claim that may be made by its manufacturer, is not guaranteed or endorsed by the publisher.
